# Bromodomain-Containing 4 Is a Positive Regulator of Interleukin-34 Production in the Gut

**DOI:** 10.3390/cells13201698

**Published:** 2024-10-14

**Authors:** Eleonora Franzè, Federica Laudisi, Rachele Frascatani, Lorenzo Tomassini, Elena De Cristofaro, Carmine Stolfi, Giovanni Monteleone

**Affiliations:** 1 Department of Systems Medicine, University of Rome “TOR VERGATA”, 00133 Rome, Italy; 2 Department of Systems Medicine, Policlinico Universitario Tor Vergata, 00133 Rome, Italy

**Keywords:** colitis, inflammatory cytokines, mucosal inflammation, JQ1

## Abstract

Experimental evidence suggests that, in the inflamed gut of inflammatory bowel disease (IBD) patients, interleukin-34 (IL-34) triggers detrimental signaling pathways. Factors/mechanisms regulating IL-34 production in IBD remain poorly characterized. Bromodomain-containing 4 (BRD4), a transcriptional and epigenetic regulator, is over-expressed in IBD, and studies in cancer cells suggest that BRD4 might positively control IL-34 expression. This study aimed to assess whether, in IBD, BRD4 regulates IL-34 expression. In IBD, there was an up-regulation of both IL-34 and BRD4 compared to the controls, and the two proteins co-localized in both lamina propria mononuclear cells (LPMCs) and epithelial cells. Flow cytometry analysis of CD45+ LPMCs confirmed that the percentages of IL-34- and BRD4-co-expressing cells were significantly higher in IBD than in the controls and showed that more than 80% of the IL-34-positive CD45-LPMCs expressed BRD4. IL-34 and BRD4 were mainly expressed by T cells and macrophages. IL-34 expression was reduced in IBD LPMCs transfected with BRD4 antisense oligonucleotide and in the colons of mice with dextran sulfate sodium-induced colitis treated with JQ1, a pharmacological inhibitor of BRD4. These data indicate that BRD4 is a positive regulator of IL-34 in IBD, further supporting the pathogenic role of BRD4 in IBD-associated mucosal inflammation.

## 1. Introduction

The etiology of ulcerative colitis (UC) and Crohn’s disease (CD), the major forms of inflammatory bowel disease (IBD), is unknown, but it has been hypothesized that these pathologies result from the interplay between genetic factors and environmental insults, which, together with changes in the intestinal microbiota and defects in counter-regulatory mechanisms, promote an excessive immune-inflammatory response and eventually intestinal damage [1,2,3]. In IBD, the lesions occur in mucosal areas infiltrated with various subsets of immune cells, which secrete several effector cytokines [4,5,6,7,8]. In this context, our previous studies showed that the involved mucosa of IBD patients contains high levels of interleukin (IL)-34 compared to the unaffected mucosa of the same patients and healthy intestines [9]. These data were consistent with results from the IBD Transcriptome and Metatranscriptome Meta-Analysis framework, which reported increased expression of IL-34 RNA transcripts in ileal samples of CD patients in comparison to the controls [10].

Like macrophage colony-stimulating factor (M-CSF-1), IL-34 signals through the colony-stimulating factor-1 receptor and regulates the survival and differentiation of monocytes and macrophages [11]. However, studies in C57/BL6 mice treated with specific M-CSF-1 or IL-34 antibodies revealed that the neutralization of M-CSF-1, but not of IL-34, reduced the number of intestinal macrophages, suggesting that M-CSF-1 is superior to IL-34 in the regulation of intestinal macrophage homeostasis [12].

In the human intestine, IL-34 targets both immune and non-immune cells and activates intracellular pathways, which contributes to the propagation of the destructive immune response [13]. Moreover, our studies demonstrated that effector cytokines that are highly synthesized in IBD (i.e., TNF) can expand IL-34 production [9]. Nonetheless, the mechanisms by which IL-34 production is regulated in IBD remain unknown.

Bromodomain-containing 4 (BRD4) is a member of the bromodomain and extra terminal domain (BET) family which binds acetylated lysine residues in both histone and non-histone proteins and controls genome activity [14,15]. BRD4 is over-expressed in the inflamed mucosal areas of patients with CD and patients with UC [16], and its down-regulation with a specific antisense oligonucleotide (AS) in IBD LPMC reduces the synthesis of TNF-α, IL-17A, and IFN-γ [16]. Moreover, a pharmacological blockade of BET proteins attenuates cytokine production and mucosal inflammation in murine models of IBD [16]. BRD4 is also a critical regulator of cancer cell growth and survival, and studies in ovarian cancer cells have recently shown that BRD4 binds the promotor region of the IL-34 gene and regulates IL-34 production [17]. It has also been shown that the co-culture of hepatoblastoma cells with macrophages induced IL-34 overexpression in cancer cells via BRD4 signaling, further supporting the role of BRD4 in the control of IL-34 expression [18].

This study aimed to assess whether BRD4 is a regulator of IL-34 production in IBD. Specifically, we assessed whether, in human IBD samples, the expression of BRD4 correlates with IL-34 content; analyzed which cells co-express the two molecules; and whether the knockdown of BRD4 in IBD LPMC is followed by a reduction in IL-34 production.

## 2. Materials and Methods

### 2.1. Patients

Biopsies were taken from the inflamed colonic mucosa of 14 patients with UC (5 females; median age: 53 years; range: 27–73 years) and 17 patients with CD (7 females; median age: 40 years; range: 24–68 years). Five CD patients and two UC patients were kept off therapy while the remaining patients received various pharmaceutical compounds (i.e., 5-aminosalicylic acid, corticosteroids, immunosuppressive, and biologics). Biopsies were also taken from the uninflamed colons of 13 healthy subjects [controls, (CTR)] (7 females; median age: 65 years; range: 47–86 years) who underwent colonoscopy for colon cancer screening. Each participant gave written informed consent, and our study was approved by the local ethics committees (Tor Vergata University Hospital, Rome (N.136.22/2022).

### 2.2. Lamina Propria Mononuclear Cell Isolation and Culture

The human LPMCs were isolated as previously described [16]. In some experiments, IBD LPMCs (5 × 10^5^) were plated into each well of a 48-well plate and transfected with a specific BRD4 AS or scrambled antisense oligonucleotide (NC AS) (both used a 2 µg/mL, Exiqon, Woburn) using Opti-MEM medium and Lipofectamine 3000 reagent according to the manufacturer’s instructions (both from Life Technologies, Milan, Italy). After 24–48 h, the cells were collected and used to extract RNA or total proteins. In additional experiments, IBD LPMCs (5 × 10^5^) were plated into each well of a 48-well plate and treated with JQ1 (200 nM, Sigma Aldrich, Milan, Italy) or Dimethyl sulfoxide (DMSO) (vehicle) (Sigma Aldrich). After 24 h, the cells were collected and used to extract RNA and analyze IL-34 by real-time PCR.

### 2.3. In Vivo Mouse Studies

The eight-week-old female Balb/c mice (Charles River Italia Srl, Calco Lecco, Italy) received either regular drinking water [control] or 1,5% dextran sodium sulfate (DSS) (MP Biomedicals, Santa Ana, CA). The mice were sacrificed on day 8, and their colon was collected for both histological examinations and RNA extraction. Additionally, mice treated with DSS received intraperitoneal injections of JQ1 (Sigma Aldrich) [50 mg/kg in 100 μL PBS per mouse] or DMSO at days 3 and 6 after the DSS administration, were killed on day 8, and had their colon collected for histology and RNA extraction. According to Italian legislation on animal experiments, the animal ethics committee were able to approve the experiments (n° 277/2023-PR).

### 2.4. Real-Time PCR

The RNA (0.5 μg/sample) was retro-transcribed into complementary DNA (cDNA), and then 1 μL of cDNA/sample was amplified using the following conditions: denaturation for 1 min at 95 °C; annealing for 30 s at 58 °C for mouse IL-34 and at 60 °C for human IL-34, human BRD4, mouse BRD4, and β-Actin; and 30 s of extension at 72 °C. Primer sequences: human BRD-4: 5′-CCTTCTACAAGCCTGTGGAC-3′ and reverse, 5′-ACTCCTGAGCATCACGGTAC-3′; human IL-34: forward 5′- ACAGGAGCCGACTTCAGTAC-3′ and reverse 5′-ACCAAGACCCACAGATACCG-3′; mouse BRD-4: 5′-CACGACTACTGTGACATCATC-3′ and reverse 5′-CTGGGCATCTCTGTACTCTC-3′; mouse IL-34: forward 5′-CACTGAGTCTGTGATGGATG-3′ and reverse 5′-CTGGTGTCAAATGATCTGGC-3′; β-actin: forward 5′-AAGATGACCCAGATCATGTTTGAGACC-3′ and reverse 5′-AGCCAGTCCAGACGCAGGAT-3′.

### 2.5. Total Protein Extraction and Western Blotting

The total proteins were extracted from the LPMCs and the human and mouse intestinal samples as previously described [16] and separated by 10% sodium dodecyl sulfate–polyacrylamide gel electrophoresis. BRD4 and IL-34 were detected using a rabbit anti-human BRD4 (final dilution: 1:1000, Novus Biological, Milan, Italy) or mouse anti-human IL-34 (final dilution: 1:1000, Abcam, Cambridge, UK), respectively, followed by horseradish peroxidase-conjugated secondary IgG monoclonal antibodies (final dilution: 1:20000, Dako, Milan, Italy). The reaction was detected with a sensitive enhanced chemiluminescence kit (Pierce, Rockford, IL, USA). After the analysis, blots were stripped and incubated with an anti-human/mouse β-actin (final dilution: 1:5000 Sigma-Aldrich). Computer-assisted scanning densitometry (Image-Lab 5.2.1, Bio-Rad Laboratories, Milan, Italy) was used to analyze the intensity of the immunoreactive bands.

### 2.6. Immunofluorescence

Immunofluorescence was performed, as previously described [16], using a rabbit anti-human BRD4 (final dilution: 1:100, Novus Biological) or mouse anti-human IL-34 (final dilution: 1:100, Abcam Cambridge, UK) and specific secondary antibodies coupled with Alexa Fluor Dyes (final dilution: 1:2000; Invitrogen, Milan, Italy).

### 2.7. Flow Cytometry Analysis

The fresh LPMCs were cultured in a complete medium with PMA (40 ng/mL) and ionomycin (1 mg/ ml) (both from Sigma-Aldrich) for 1 h followed by the addition of brefeldin A (10 mg/mL) and monensin (10 mg/mL) (both from eBioscience, San Diego, CA, USA) for a further 3 h, and a LIVE/DEAD cell viability assay was then performed according to the manufacturer’s instructions (Life Technologies). The non-specific binding of the monoclonal antibodies was prevented by pre-incubating the cells with an Fc receptor-blocking antibody (final dilution: 1:100 eBioscience) for 15 min at room temperature. The cells were then washed and stained with the following monoclonal anti-mouse antibodies: CD45-PeCy7, CD3-APC-H7, CD14-FITC (final dilution: 1:50, Miltenyi Biotech, Bologna, Italy), CD19- FITC, CD16-PerCP, CD56–V450, and CD11c-PerCP (final dilution: 1:50, Becton Dickinson, Milan Italy), or respective isotype control antibodies. Afterward, the cells were fixed with a Transcription Factor Staining buffer set (eBioscience) and permeabilized using Permeabilization buffer (eBioscience). Cells were stained intracellularly with anti-human IL-34-Pe (1:50 final dilution, Minneapolis, MN, USA) and anti-human BRD4-APC (1:50 final dilution, Abcam, Cambridge, UK) and CD68- APC-H7 (1:50 final dilution, Miltenyi Biotech, Bologna, Italy) or relative isotype control antibodies. The fluorescence signals were collected by using a FACSVerse (Becton Dickinson) and analyzed by FlowJO V10 (Becton Dickinson).

### 2.8. Statistical Analysis

Student’s t-test was used to ascertain differences between groups, and Pearson’s test to correlate IL-34 and BRD4 protein expression in IBD samples. All the analyses were performed using Graph-Pad 6 software.

## 3. Results

### 3.1. IL-34 and BRD4 Are Over-Expressed in IBD

**Figure 1:**
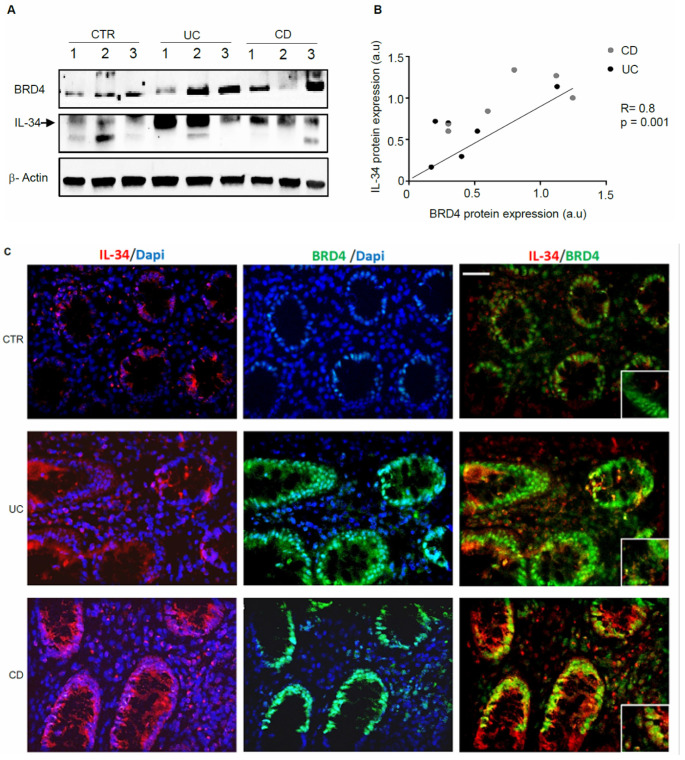
In IBD, BRD4 expression correlates with interleukin (IL)-34 content. (**A**). Representative Western blots showing BRD4, IL-34, and β-actin in total proteins extracted from colonic samples of 3 controls (CTR), 3 UC patients, and 3 CD patients. The example is representative of 2 experiments in which 6 CTRs, 6 UC patients, and 6 CD patients were analyzed. Note that one CD patient and one UC patient had similar levels of BRD4 and IL-34. (**B**). Correlation between the protein expression of BRD4 and IL-34 in UC patients (black circles) and CD patients (grey circles) as evaluated by Western blotting of mucosal samples (*p* = 0.001, Pearson’s test coefficient r = 0.8). (**C**). Representative images of immunofluorescence staining of colon sections taken from 1 CTR, 1 patient with UC, and 1 patient with CD, and analyzed for the expression of BRD4 (green), IL-34 (red) and DAPI (blue) (20× magnification). The example is representative of 3 separate experiments in which sections of 3 colonic CTRs, 3 UC patients, and 3 CD patients were analyzed. The lower right inset shows higher magnification (40×).

We firstly assessed IL-34 and BRD4 protein expression in mucosal samples taken from active IBD patients and controls. In line with our previous studies [9,16], a marked up-regulation of both IL-34 and BRD4 was seen in biopsy samples of patients with UC and patients with CD in comparison to the controls, while there was no apparent difference between CD and UC (Figure 1A and Figure S1). Additionally, in IBD mucosal samples, there was a positive correlation between BRD4 and IL-34 expression (Figure 1B). Immunofluorescence staining of IBD and control biopsies confirmed the up-regulation of both IL-34 and BRD4 in IBD and showed a co-localization of the two proteins in both epithelial cells and LPMCs (Figure 1C).

### 3.2. The Fractions of IL-34 and/or BRD4-Expressing LPMCs Are Increased in IBD

**Figure 2:**
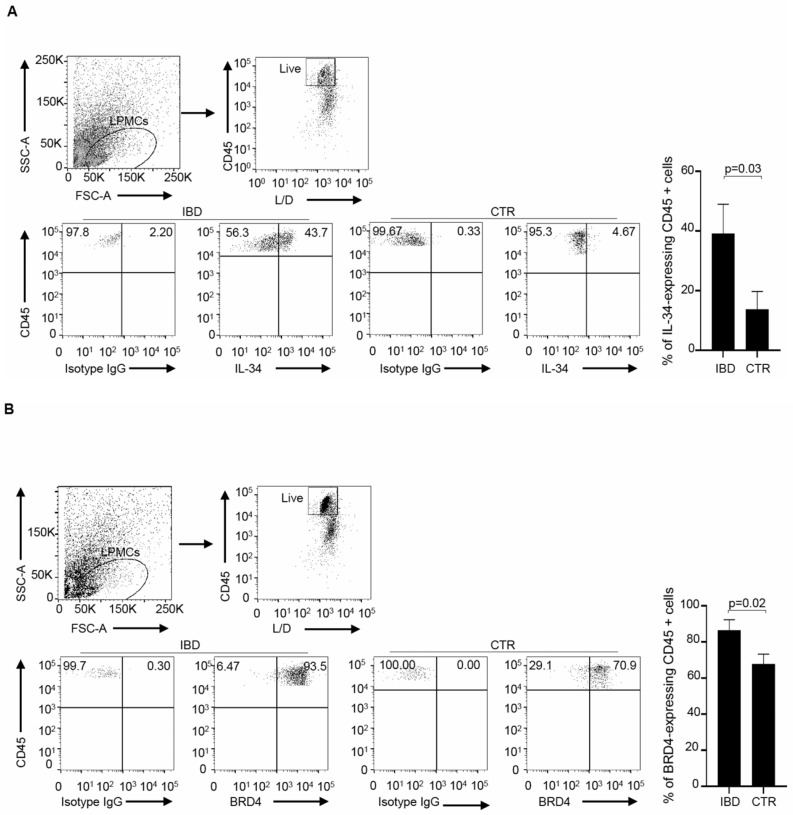
The percentages of live CD45+LPMCs expressing IL-34 or BRD4 are higher in IBD than in controls. (**A**,**B**). LPMCs were isolated from 5 IBD patients (3 UC and 2 CD patients) and 4 CTRs and analyzed by flow cytometry for IL-34 (**A**) or BRD4 (**B**). The positive cells were selected in the gate of live CD45+LPMCs. Staining results with an isotype control antibody for IL-34 (**A**) or BRD4 (**B**) are also shown. The right panels indicate the mean ± SEM of the percentages of IL-34 or BRD4-expressing CD45+ LPMCs.

**Figure 3:**
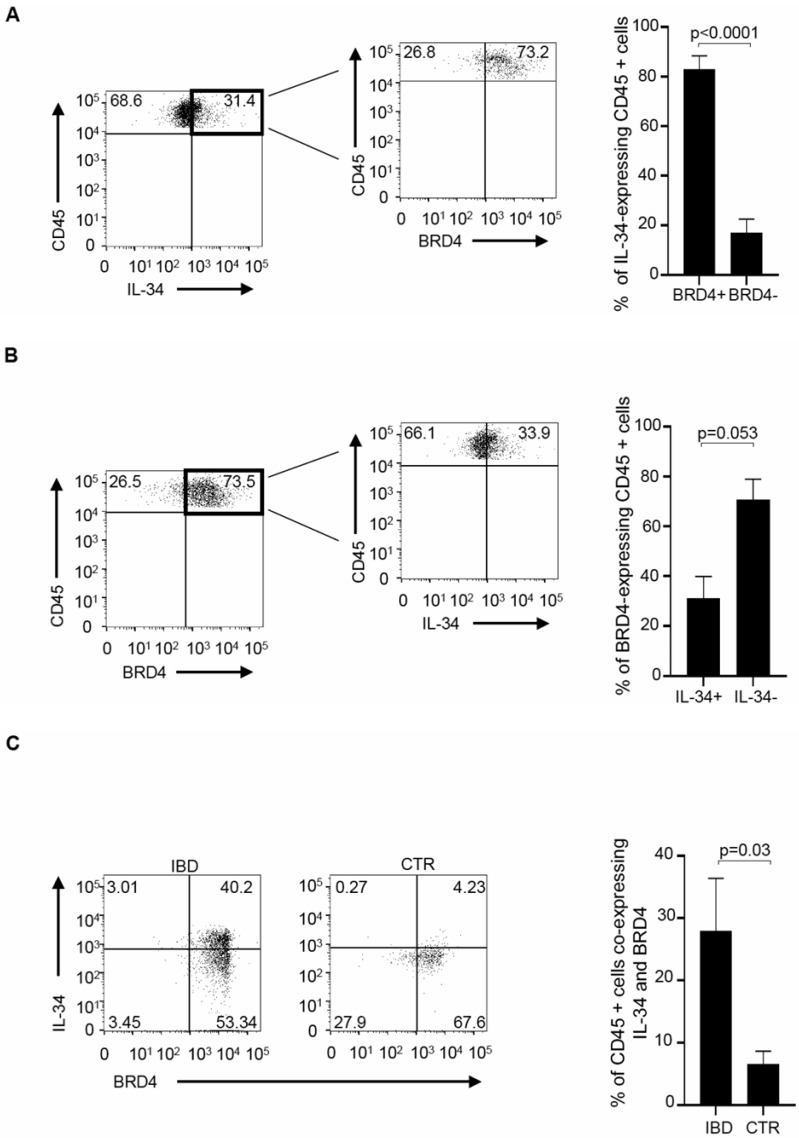
In IBD, most of the IL-34-positive CD45+LPMCs express BRD4, and the percentages of CD45+ LPMCs co-expressing IL-34 and BRD4 are significantly higher than in the controls. (**A**). LPMCs were isolated from 5 IBD patients (3 UC and 2 CD patients) and analyzed by flow cytometry for IL-34 and BRD4. The gated IL-34-expressing live CD45+ LPMCs were analyzed for BRD4 expression. One of five representative experiments is shown. The right panel indicates the mean ± SEM of the percentages of IL-34-expressing CD45+ LPMCs either positive or not for BRD4. (**B**). LPMCs were isolated and analyzed as above. The gated BRD4-expressing live CD45+ LPMCs were analyzed for IL-34 expression. One of five representative experiments is shown. The right panel indicates the mean ± SEM of the percentages of BRD4-expressing CD45+ LPMCs either positive or not for IL-34. (**C**). LPMCs were isolated from 5 IBD patients (3 UC and 2 CD patients) and 4 CTRs and analyzed by flow cytometry for the expression of BRD4 and IL-34 in the gate of live CD45+ cells. The right panel indicates the mean ± SEM of the percentages of CD45+ LPMCs co-expressing BRD4 and IL-34 in IBD and controls.

Having shown that LPMCs co-express IL-34 and BRD4, we next assessed which immune cell types are positive for the two proteins. By flow cytometry, CD45+ cells were gated among the live LPMCs population and evaluated for the expression of IL-34 and/or BRD4. The percentages of IL-34- or BRD4-expressing cells were significantly higher in IBD than in the controls (Figure 2A,B). Moreover, in IBD, more than 80% of the IL-34-positive CD45+LPMCs expressed BRD4 (Figure 3A). In contrast, less than 40% of BRD4-positive CD45+LPMCs expressed IL-34 (Figure 3B).

Consistently, the fractions of CD45+ LPMCs co-expressing BRD4 and IL-34 were significantly higher in IBD than in the controls (Figure 3C).

### 3.3. IL-34 and BRD4 Are Co-Expressed by Multiple Immune Cells in IBD

**Figure 4:**
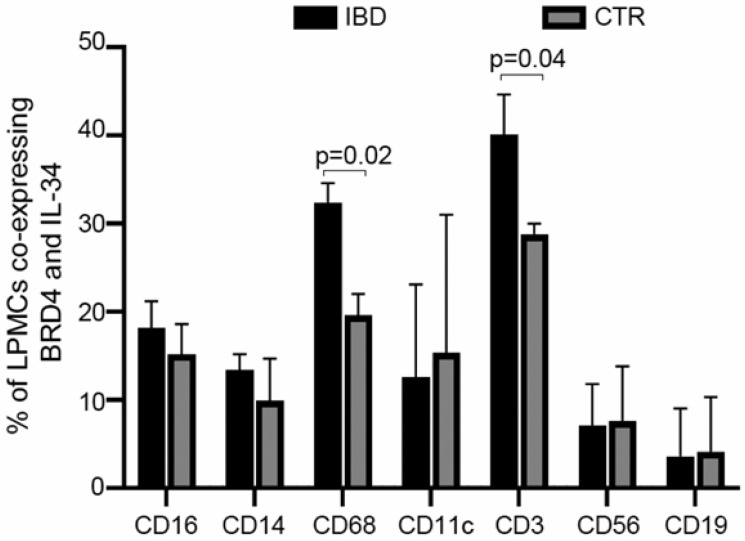
IL-34 and BRD4 are co-expressed by several immune cells in both CTR and IBD patients. LPMCs were isolated from 5 IBD patients (3 UC and 2 CD patients) and 4 CTRs and analyzed for the expression of CD16, CD14, CD68, CD11c, CD3, CD56, and CD19 in live CD45+ cells expressing BRD4 and IL-34. The data indicate mean ± SEM of 5 IBD samples and 4 CTR samples.

To ascertain which immune cells express IL-34 and/or BRD4, IBD and control LPMCs were stained with IL-34, BRD4, and markers of immune cells and analyzed by flow cytometry. Although in both IBD and controls, IL-34 and BRD4 were co-expressed by multiple immune cell types, the fractions of CD3+ and CD68+ LPMCs co-expressing the two proteins were significantly greater in IBD than in the controls (Figure 4).

### 3.4. BRD4 Inhibition Reduces the Intestinal Expression of IL-34

**Figure 5:**
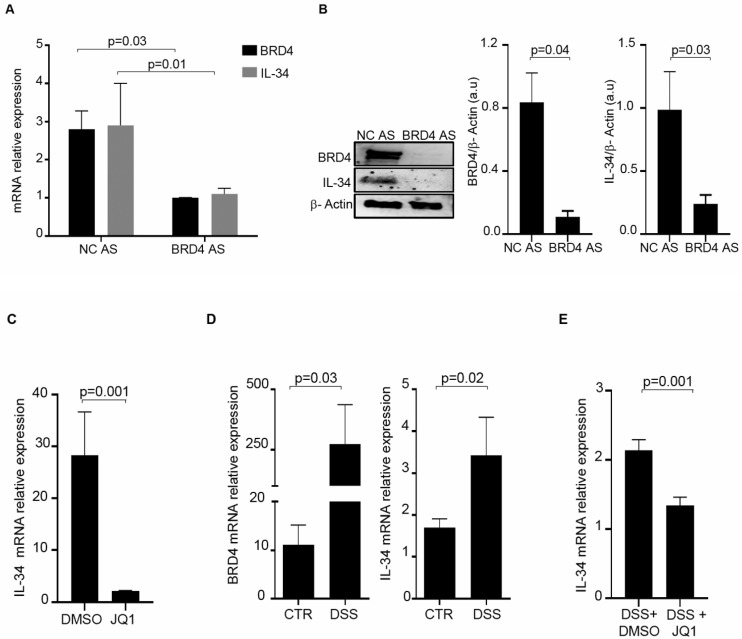
BRD4 inhibition reduces the expression of IL-34. (**A**). IBD LPMCs were transfected with either a scrambled control oligonucleotide (NC AS) or BRD4 AS for 24 h and BRD4 (black) and IL-34 (grey) RNA transcripts were analyzed by real-time PCR. Levels were normalized to β-actin. Data indicate mean ± SEM of 4 independent experiments. (**B**). IBD LPMCs were transfected with either NC AS or BRD4 AS for 48 h and BRD4, IL-34, and β-actin were analyzed by Western blotting. One of 4 independent experiments is shown. The right panels show the quantitative analysis of the BRD4/β-actin ratio and IL-34/β-actin ratio in protein extracts as measured by densitometry scanning of Western blots. Values are expressed in arbitrary units (a.u.) and indicate the mean ± SEM of all experiments. (**C**). IBD LPMCs were treated with JQ1 (200 nM) or vehicle (DMSO) for 24 h, and IL-34 RNA transcripts were analyzed by real-time PCR. Levels were normalized to β-actin. The data indicate mean ± SEM of 4 independent experiments. (**D**). Mice received either regular drinking water (CTR) or dextran sulfate sodium (DSS) and were killed on day 8. BRD4 and IL-34 mRNA expression was evaluated in colonic tissue by real-time PCR and levels were normalized to β-actin. The data indicate mean ± SEM of 6 CTR mice and 9 DSS-treated mice. (**E**). Mice receiving DSS were intraperitoneally given JQ1 (DSS+JQ1) or vehicle (DMSO) on days 3 and 6 and then killed on day 8. IL-34 mRNA expression was evaluated in colonic tissue by real-time PCR and levels were normalized to β-actin. The data indicate mean ± SEM of all samples.

To evaluate whether IL-34 is under the control of BRD4, IBD LPMCs were transfected with a control or BRD4 AS, and then the RNA and protein content of BRD4 and IL-34 was evaluated by real-time PCR and Western blotting, respectively. Knockdown of BRD4 significantly diminished IL-34 RNA expression (Figure 5A). Densitometry analysis of Western blots confirmed the down-regulation of IL-34 in BRD4-deficient IBD LPMCs (Figure 5B and Figure S2). Moreover, IBD LPMCs were treated with JQ1, a pharmacological inhibitor of the BET protein, and IL-34 was then evaluated by real-time PCR. JQ1 treatment significantly reduced IL-34 RNA expression (Figure 5C). To further confirm the positive regulation of BRD4 on IL-34 expression in the gut, we analyzed the expression of the two proteins in the colons of mice with DSS-induced colitis. The colons of mice receiving DSS exhibited greater levels of both IL-34 and BRD4 than control mice (Figure 5D). In vivo administration of JQ1 to colitic mice significantly reduced IL-34 RNA expression (Figure 5E).

## 4. Discussion

This study was undertaken to assess whether BRD4 controls IL-34 expression in IBD because there is evidence that both these proteins are up-regulated in the inflamed intestine of IBD patients [9,16], and studies in tumor cells have shown the ability of BRD4 to positively regulate IL-34 expression [17,18]. In this paper we confirm that, in the inflamed tissue of patients with IBD, there are elevated levels of both BRD4 and IL-34, and we show that the two proteins are co-expressed in both epithelial cells and LPMCs. Characterization of the cell source of these two proteins revealed that multiple immune cells co-express IL-34 and BRD4 in both the normal and inflamed gut. Nonetheless, the fractions of T cells and macrophages co-expressing the two proteins were significantly higher in IBD than in controls. When the analysis was restricted to IL-34-expressing LPMCs isolated from IBD, it was evident that more than 80% of cells were positive for BRD4, further supporting the hypothesis that BRD4 could be involved in the regulation of IL-34 production. Indeed, the knockdown of BRD4 with a specific AS in IBD LPMCs was followed by a marked down-regulation of IL-34 expression. A similar finding was seen when IBD LPMCs were treated with JQ1, a potent and selective pharmacological inhibitor of the BRD4 signaling pathway, thus excluding the possibility that the reduced expression of IL-34 in BRD4 AS-transfected IBD LPMCs is due to off-target effects of the AS. We have recently shown that mice with DSS-colitis given JQ1 exhibit attenuation of the ongoing mucosal inflammation, which was paralleled by a reduction in the percentages of both CD3+T cells and CD11B+ CD11c+ cells and a significant reduction in RNA transcripts for TNF-α, IL-6, IL-17A, and IFN-γ, without affecting IL-10 RNA expression [16]. Further analysis of such samples revealed that the induction of DSS-colitis was associated with enhanced expression of both IL-34 and BRD4, and samples extracted from colonic specimens of mice treated with JQ1 had a significant reduction in RNA transcripts for IL-34. Our data confirm and expand on results from previous studies showing that JQ1 inhibits IL-34 expression in the murine ovarian cancer cell lines OV3121-RAS4 and HM-1, and this effect was not due to changes in cell viability [17,18]. Rather, in the same cell cultures, JQ1 treatment reduced BRD4 binding to the promoter region of the Il-34 gene, thus implying a direct, positive regulation of IL-34 expression by BRD4. The fact that nearly one-fifth of IL-34-positive IBD LPMCs do not express BRD4 suggests the involvement of additional factors in the regulation of IL-34. NF-kB and Specificity Protein 1 could be some of these factors, as supported by studies in other systems [19,20,21].

IL-34, initially described as a main regulator of monocyte cell survival and differentiation, controls several pathways that amplify the IBD-associated pathogenic inflammatory response. For instance, we have recently shown that IL-34 can target both immune cells and stromal cells and stimulate the secretion of inflammatory cytokines and collagen [9,22]. Our previous studies also showed that cytokines over-produced in IBD, such as TNF-α, can enhance the production of IL-34 and, consistently, the neutralization of TNF with infliximab, a TNF blocker, reduced IL-34 production in IBD LPMC cultures, and in vivo in IBD patients [9]. Notably, TNF-α also positively regulates BRD4 [6], raising the possibility that TNF can induce IL-34 through a BRD4-dependent mechanism. The data of the present study add a further piece of evidence to the mechanisms regulating IL-34 production in IBD.

Because BRD4 is a master regulator of the production of multiple inflammatory cytokines, it is logical to hypothesize that BRD4 inhibitors are more efficient than IL-34 blockers in the control of gut inflammation. This hypothesis is supported by the therapeutic effects of JQ1 in mice with DSS- and TNBS-induced colitis [16], as well as by the ability of the small molecule MS402, another selective inhibitor of the first bromodomain of BET proteins, to ameliorate T cell transfer-induced colitis in mice [23]. In this context, it is noteworthy that studies in rats and mice deficient in IL-34 have documented the enhanced production of multiple auto-antibodies and increased susceptibility to DSS- and TNBS-colitis, probably reflecting defects in regulatory cells (Tregs) [24]. Indeed, IL-34-deficient CD4+ Tregs are unable to protect immunodeficient rats from a wasting disease induced by the transfer of pathogenic cells, in contrast to IL-34-expressing CD4+ Tregs [24]. These IL-34-induced divergent effects on the course of pathological inflammation are not seen exclusively in the gut, as similar findings were documented in rheumatoid arthritis (RA). While a growing body of evidence supports the role of IL-34 as a driver of RA pathogenesis, some recent studies showed that IL-34 alleviates synovial inflammation, potentially by inducing regulatory Tregs [25,26,27,28]. It is thus conceivable that IL-34 can exert both regulatory and inflammatory functions, depending on the tissue context in which the cytokine acts.

We are aware that this study has some limits. Particularly, the analysis was performed in a relatively small group of patients, and biopsy samples were taken at a single time point in the natural history of the disease. Therefore, these data deserve further confirmation in larger studies assessing both IL-34 and BRD4 during the different evolutive phases of the disease. The current data do not help us to understand whether the assessment of IL-34/BRD4 content in IBD biopsy samples could be useful in the management of these patients nor whether the activation of the BRD4/IL-34 axis can influence the responsiveness to specific drugs used in IBD. We would also like to point out that the controls of this study were slightly older than the IBD patients, but it is unlikely that this could influence the results because our previous studies showed that active mucosal inflammation is the only determinant in the induction of both proteins [9,16].

In conclusion, our study shows that BRD4 is a positive regulator of IL-34 production in inflamed tissue of IBD patients. These findings, together with the recent demonstrations that BRD4 inhibitors are therapeutic in mouse models of IBD-like colitis, suggest that BRD4 could be a promising target for IBD therapy.

## Data Availability

The original contributions presented in the study are included in the article, further inquiries can be directed to the corresponding author.

## Supplementary Materials

The following supporting information can be downloaded at: https://www.mdpi.com/article/10.3390/cells13201698/s1, Figure S1. Uncropped western blots showing BRD4 (A), IL-34 (B), and β-Actin (C) in mucosal samples taken from 2 normal controls, 3 patients with ulcerative colitis (UC), and 3 patients with Crohn’s disease (CD). These data were used to prepare the Figure 1A. Figure S2. Uncropped western blots showing BRD4 (A), IL-34 (B) and β-Actin (C) in IBD LPMC transfected with a control (NC) or BRD4 antisense oligonucleotide AS). These data were used to prepare the Figure 5B.
