# Supplementary material for: Bromodomain-Containing 4 Is a Positive Regulator of Interleukin-34 Production in the Gut
Source: Cells. 2024 Oct 14;13(20):1698. doi: 10.3390/cells13201698 (PMC11505644; doi:10.3390/cells13201698)
Supplement: Supplementary file 1 [file cells-13-01698-s001.zip › cells-3213465-supplementary.pdf]

cells

Supplementary Material

Bromodomain-Containing 4 Is a Positive Regulator of Interleukin-34 Production in the Gut

Eleonora Franzè [1], Federica Laudisi [1], Rachele Frascatani [1], Lorenzo Tomassini [1], Elena De Cristofaro [2], Carmine Stolfi [1] and Giovanni Monteleone [1,*]

[1] Department of Systems Medicine, University of Rome "TOR VERGATA", Rome 00133, Italy
[2] Department of Systems Medicine, Policlinico Universitario Tor Vergata, Rome 00133, Italy
* Correspondence: gi.monteleone@med.uniroma2.it

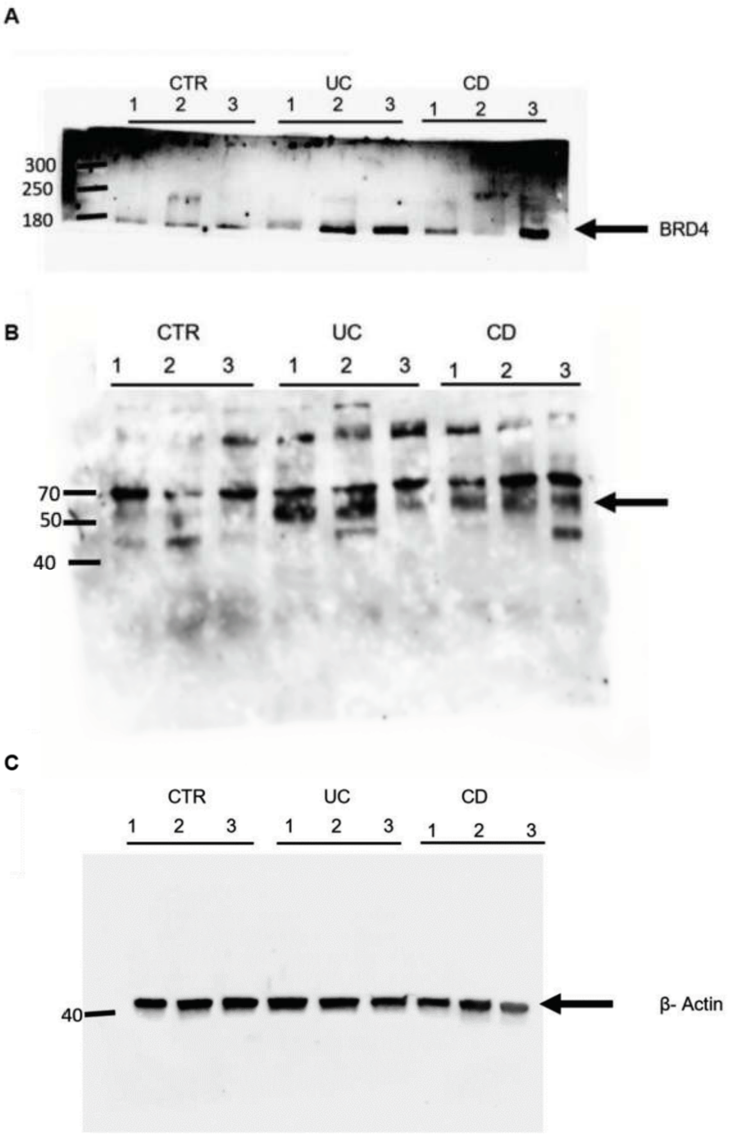

Figure S1. Uncropped western blots showing BRD4 (A), IL-34 (B), and β-Actin (C) in mucosal samples taken from 2 normal controls, 3 patients with ulcerative colitis (UC), and 3 patients with Crohn's disease (CD). These data were used to prepare the Figure 1A.

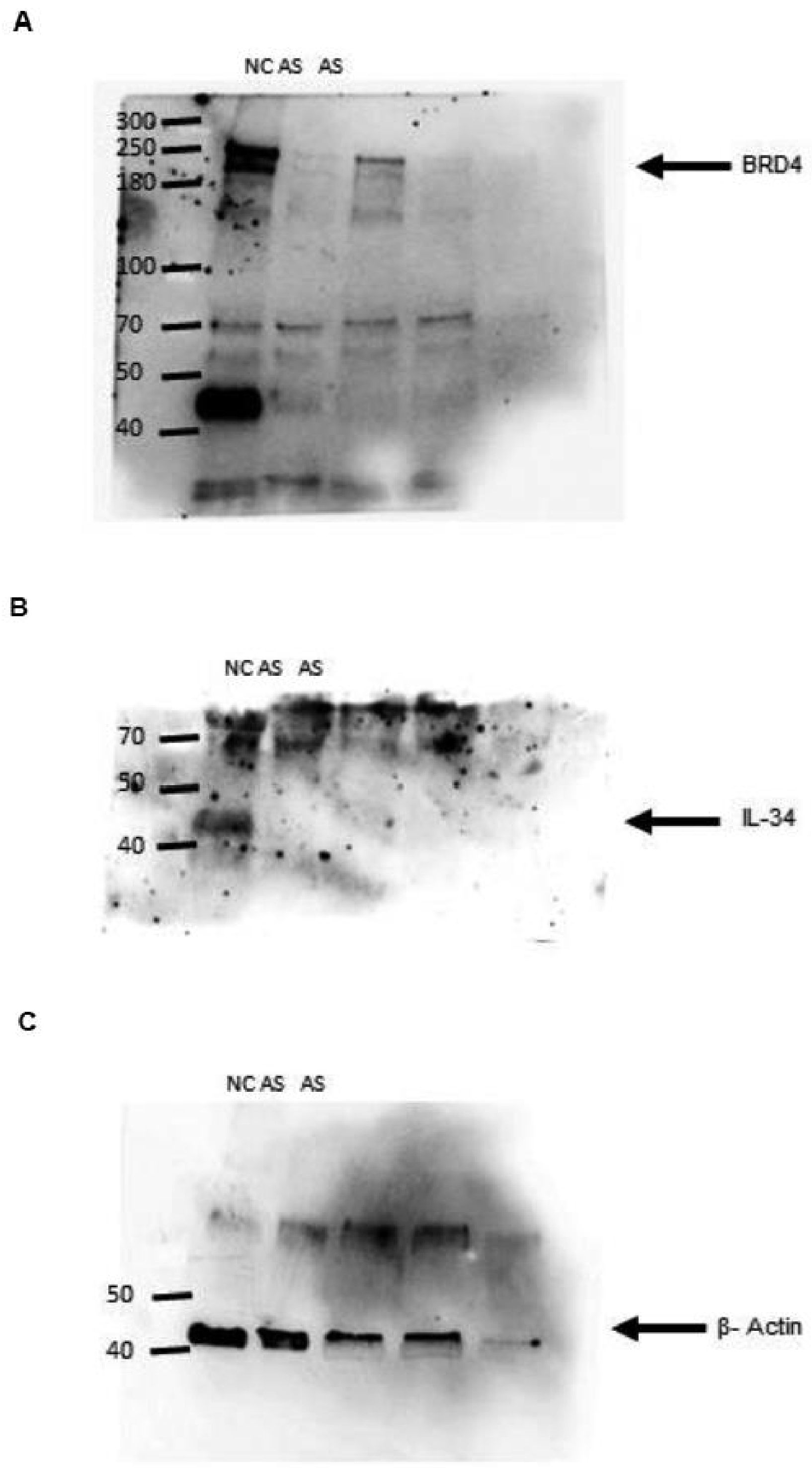

Figure S2. Uncropped western blots showing BRD4 (A), IL-34 (B) and β-Actin (C) in IBD LPMC transfected with a control (NC) or BRD4 antisense oligonucleotide AS). These data were used to prepare the Figure 5B.
